# Anti-apoptotic treatment of warm ischemic male rat livers in machine perfusion improves symptoms of ischemia-reperfusion injury

**DOI:** 10.21203/rs.3.rs-3260870/v1

**Published:** 2023-08-22

**Authors:** Mohammadreza Mojoudi, McLean S. Taggart, Anil Kharga, Huyun Chen, Antonia T. Dinicu, Benjamin T. Wilks, James F. Markmann, Mehmet Toner, Shannon N. Tessier, Heidi Yeh, Korkut Uygun

**Affiliations:** Center for Engineering in Medicine and Surgery, Department of Surgery, Massachusetts General Hospital; Center for Engineering in Medicine and Surgery, Department of Surgery, Massachusetts General Hospital; Center for Engineering in Medicine and Surgery, Department of Surgery, Massachusetts General Hospital; Center for Engineering in Medicine and Surgery, Department of Surgery, Massachusetts General Hospital; Center for Engineering in Medicine and Surgery, Department of Surgery, Massachusetts General Hospital; Center for Engineering in Medicine and Surgery, Department of Surgery, Massachusetts General Hospital; Center for Transplantation Sciences, Department of Surgery, Massachusetts General Hospital; Center for Engineering in Medicine and Surgery, Department of Surgery, Massachusetts General Hospital; Center for Engineering in Medicine and Surgery, Department of Surgery, Massachusetts General Hospital; Center for Transplantation Sciences, Department of Surgery, Massachusetts General Hospital; Center for Engineering in Medicine and Surgery, Department of Surgery, Massachusetts General Hospital

## Abstract

Liver donation after cardiac death (DCD) makes up a small percentage of the donor pool and poses a higher risk of graft loss compared to donation after brain death (DBD); this is a result of ischemia reperfusion for which the exact injury mechanisms are currently not fully understood. However, reperfusion injury has been shown to lead to necrosis as well as apoptosis at the cellular level. In this work, we propose that use of the pro-survival, anti-apoptotic CEPT cocktail in post-ischemia normothermic machine perfusion (NMP) may improve recovery in rat livers subjected to extended durations of warm ischemia. Livers procured from male lewis rats were subjected to 90 minutes of warm ischemia, followed by 6 hours of NMP where they were treated with the survival-enhancing anti-apoptotic cocktail (CEPT), the vehicle (DMSO) or the base media with no additives. The CEPT-treated group exhibited lower expression of hepatic injury biomarkers, and improvement in a range of hepatocellular functions associated with the hepatic parenchyma, biliary epithelium and especially the sinusoidal endothelium. This study’s findings provide useful insight for further investigation of the extent of apoptotic contribution to ischemia reperfusion injury (IRI).

## Introduction

The growing use of novel ex-vivo methods of graft viability assessment such as normothermic machine perfusion (NMP) over standard static cold storage (SCS) has been shown to lead to an increase in overall graft survival as well as liver-recipient matching by expanding the transplantation criteria to include donation after cardiac death (DCD)^1,2^. However, despite the increased use of such marginal livers, there remains a notable gap between transplant demand and donor availability, which can potentially be narrowed by addressing the underlying mechanisms of injury in livers severely damaged by warm ischemia^3^.

DCD donors are often rejected for transplantation and make up a small percentage of the donor pool due to a significantly higher risk of early graft dysfunction compared to donors after brain death (DBD)^4^. This is primarily a result of ischemia-reperfusion injury (IRI), a cascade of acute inflammatory responses initiated by injured hepatic cells upon restoration of circulation within the graft. While the mechanisms involved in this phenomenon are not fully understood, it is known that IRI triggers several cell death pathways of not only the inflammatory kind including necrosis, necroptosis, pyroptosis and ferroptosis, but also apoptosis^5^, suggesting interfering cell death pathways may be a practical means of improving overall liver recovery following IRI. Previous studies have validated the potential of this hypothesis by demonstrating that inhibition of hepatocellular death through caspase inhibition results in lower expression of inflammatory molecules, amelioration of sinusoidal dysfunction and improved hepatocyte phenotype in chronically diseased rat livers^6^; however, the effects of such treatments on IRI in liver transplantation remains relatively understudied.

To explore the extent of apoptotic contribution to the effects of IRI in liver, we developed a protocol for recovery of male rat livers subjected to 90 minutes of warm ischemia (WI) at 37°C, where during 6 hours of post-ischemia NMP they were treated with CEPT, a survival-enhancing anti-apoptotic cocktail consisting of Chroman 1, Emricasan, Polyamines and Trans-ISRIB, previously shown to enhance the viability of human pluripotent stem cells (hPSCs) and the resulting differentiated cells^7^. Knowing that the effects of CEPT are currently understudied in the context of machine perfusion, we aim to specifically explore its efficacy in halting the exacerbation of cellular injury following ischemia-reperfusion and facilitating restoration of tissue functionality.

## Methods

### Ethics Declarations

All procedures reported below are in accordance with ARRIVE guidelines, as well as the guidelines established in the experimental protocol approved by the Institutional Care and Use Committee (IACUC) of MGH (Protocol Number 2017N000227).

### Experimental design

**Figure 1:**
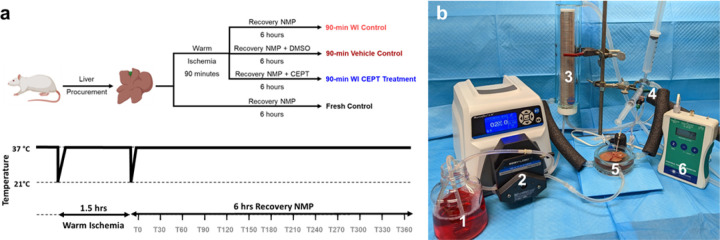
Experimental design. (a) Schematic illustration of the experimental design and groups (n=5 per group) (b) Perfusion setup consisting of: 1. Perfusion media 2. Digital Peristaltic Pump 3. Oxygenation chamber 4. Bubble trap 5. Perfusion basin 6. Pressure monitor

As depicted in Fig. 1a, whole livers were procured from adult Lewis rats and underwent four conditions (n = 5 for each group): (1) Fresh Control: Immediate connection to machine perfusion device (2) 90-min warm ischemia (WI) CEPT Treatment (3) 90-min WI Vehicle (DMSO) Control (4) 90-min WI Control. WI was carried out with 1.5 hours of storage in a bag of 50 mL saline, held at 37°C. All groups followed with 6 hours of NMP at 37°C. The CEPT cocktail or DMSO were added directly to the NMP base perfusate.

### Perfusate Preparation

Recovery perfusate was composed from a base of 500 mL Williams’ Medium E (with sodium bicarbonate, without L-glutamine, with phenol red) (Sigma-Aldrich, St. Louis, MO, USA) into which 5 g polyethylene glycol 35,000 (PEG) (Sigma-Aldrich), 12 mg dexamethasone (water-soluble) (Sigma-Aldrich), 5 g bovine serum albumin (Sigma-Aldrich), 400 mg sodium bicarbonate (Sigma-Aldrich),, 5 mL heparin (MGH Pharmacy), and 2 mL penicillin-streptomycin (Thermo Fisher Scientific, Waltham MA, USA) were added. On the day of experiment, 5 mL L-glutamine (Sigma-Aldrich), 50 uL insulin (MGH Pharmacy), 100 uL hydrocortisone (MGH Pharmacy) and 768 mg L-glutathione (Sigma-Aldrich) were added directly to perfusate before beginning perfusion. For the CEPT perfusate, 50 nM chroman 1 (MedChemExpress, Monmouth Junction NJ, USA), 5 uM Emricasan (MedChemExpress), 0.7 uM trans-ISRIB (Tocris Bioscience), 40 ng/mL putrescine (Sigma-Aldrich), 4.5 ng/mL spermidine (Sigma-Aldrich), and 8 ng/mL spermine (Sigma-Aldrich), all suspended in dimethyl sulfoxide (DMSO) (Sigma-Aldrich) were added directly to perfusate before beginning perfusion; the concentrations were identical to those of the original CEPT cocktail. For vehicle perfusate, 800 uL DMSO was added directly to perfusate before beginning perfusion.

### Liver Procurement

Livers from adult, male Lewis rats (250–300 g) (Charles River Laboratories, Boston MA, USA) were elected to be used for all experiments, after preliminary testing of the study design with female rats of the same strain and weight proved to be too damaging to the livers to allow for any recovery after the proposed ischemic duration, jeopardizing the unbiased comparability of results among different groups. In total, livers from 3 female rats underwent the experimental design, representative data for which can be found in supplementary Fig. 1. The basis of this difference will be explored in future studies. All animals were socially housed in controlled, standard conditions (21°C, 12-hour light/dark cycle, 30–70% humidity, mixed paper/cellulose bedding, pathogen-free HEPA filtered ventilated cages). The rats had unrestricted access to sterile water and chow, in accordance with National Research Council Guidelines. Rats were cared for by the Massachusetts General Hospital (MGH) Center for Comparative Medicine (CCM).

Livers were procured from male Lewis rats (250–300g; age 10–12 weeks; Charles River Laboratories, Wilmington, MA, USA). Donor rats were anaesthetized under 5% isoflurane. The abdomen was opened via a transverse abdominal incision. Ligaments connecting the superior and inferior portions of the liver were dissected and the portal vein was exposed. The gastric and splenic branches of the portal vein, as well as the hepatic artery were ligated using 6 − 0 silk. The bile duct was partially dissected, cannulated using 24g tubing, and secured with 6 − 0 silk. The inferior vena cava was heparinized with 1 U/g using a 30-gauge insulin syringe. Following 5 minutes of heparin circulation, the portal vein was cannulated with a 16g cannula, followed by transection of the IVC. The cannula was connected to 16g tubing attached to a 50mL syringe containing 1mL heparin in 60mL saline. The portal vein was hand flushed at 10mL/min for 4 minutes, after which the remaining connective tissue was dissected, and the liver was removed from the body cavity. Following removal, the liver was flushed with the remaining 20mL saline, immediately weighed, and connected to the perfusion system, keeping warm ischemic time below 5 minutes.

### Normothermic Machine Perfusion

Perfusate circulation was carried out using a roller pump system (Masterflex L/S, Vernon Hills, IL) with two separate sets of tubing delivering perfusate into and out of the 500 mL perfusion reservoir (Fig. 1b). The system was consistently kept at a temperature of 37°C via a water bath (PolyScience, Niles, Illinois, USA) continuously pumping heated water through the double-jacketed perfusion system components (Radnoti, Covina, CA, USA). Perfusate oxygen concentration was maintained within a close range of 500 mmHg using a 95% O2/5% CO2 gas cylinder (Airgas, Radnor, PA, USA). System pressure was zeroed, the liver was placed in the tissue bath and connected to the system. The flow rate was brought from 5mL/min to 30mL/min gradually, maintaining a maximum portal pressure of 5mmHg. Outflow samples were collected every 30 minutes from the IVC, while inflow samples were collected from a port placed above the cannula perfusing the portal vein. The liver was weighed upon the end of perfusion to determine weight change. Biopsies of the left lateral lobe (LLL) and right medial lobe (RML) were carried out immediately after, with the LLL sample being snap frozen in liquid nitrogen for subsequent ATP analysis and the RML sample being formalin-fixed for histological analysis.

### Liver Viability Assessment

Every 30 minutes, inflow and outflow perfusate samples were analyzed using a Siemens Rapidpoint 500 (Siemens, Munich, Germany). Liver performance metrics were analyzed to determine liver functionality during perfusion (pH, O2 consumption, lactate clearance, potassium). Following perfusion, hourly outflow samples were analyzed for hepatic injury markers (AST and ALT enzymes) using a Piccolo Xpress (Abaxis, Union City, CA, USA). Portal resistance was determined using pressure readings taken every half hour, and defined as pressure divided by flow, normalized to weight (g). Oxygen consumption was defined as portal inflow pO2 minus IVC outflow pO2. Weight change was defined as final weight minus initial weight divided by initial weight.

### Histological Analysis

Liver tissue samples were sectioned and stained with hematoxylin-eosin (H&E) and Terminal deoxynucleotide transferase dUTP nick end labeling (TUNEL) (Specialized Histopathology Services Core, MGH, Charlestown, MA, USA).

### Caspase 3/7 Activity Assay

For caspase 3/7 activity measurements, livers were sacrificed at the end of each perfusion and 300–500 mg of the livers were flash-frozen in liquid nitrogen for − 80°C storage. On the day of measurement, the frozen livers were added to ice-cold PBS at 50 mg/mL and prepared for tissue homogenization (n = 3 per treatment group) using the gentleMACS Dissociator (Miltenyi Biotec, Bergisch Gladbach, Germany). Then, equal volume of room-temperature PBS was added to the lysate to dilute to 25 mg/mL and 100 uL of the diluted lysate was treated with room-temperature Caspase-Glo 3/7 luminescent assay (Promega, Madison, Wisconsin, USA), adding 1:1 volume of caspase-glo to liver lysate in a 96-well white-bottom plate. The plate was mixed using a plate shaker set to 250 rpm for 30 seconds and then left to incubate at room temperature for 30 minutes. Following incubation, the luminescence was read using a plate reader.

### Statistical Analysis and Illustrations

Statistical analysis was performed with Prism 8 software Version 9.1.2 (Graphpad Software, San Diego, CA, USA, graphpad.com) with a two-sided significance level of 0.05. Two-way analysis of variance (ANOVA) was performed to compare time-dependent perfusion data, followed by Tukey’s post-hoc test to examine statistical difference. Perfusion metrics were reported as means, with standard deviation as error bars. Statistical analysis was performed with Prism 8 software Version 9.1.2 (Graphpad Software, San Diego, CA, USA, graphpad.com) with a two-sided significance level of 0.05. Two-way analysis of variance (ANOVA) was performed to compare time-dependent perfusion data, followed by Tukey’s post-hoc test to examine statistical difference. Perfusion metrics were reported as means, with standard deviation as error bars. All illustrations were created with Biorender (Toronto, ON, Canada).

## Results

### Liver Viability during Recovery Perfusion

**Figure 2:**
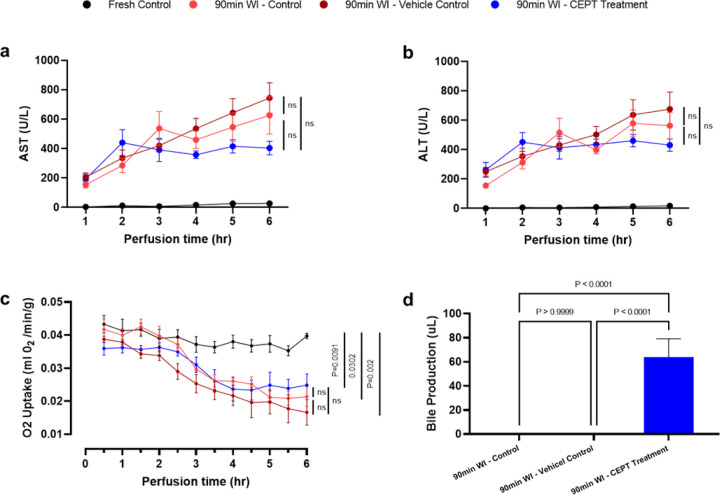
Liver viability following 1.5 hours of ex-vivo warm ischemia. Concentrations of (a) Aspartate aminotransferase (AST) and (b) Alanine transaminase in perfusate samples collected hourly from the IVC. Lower concentrations near the end of the 6-hour NMP suggest higher rates of recovery from ischemic damage in livers treated with CEPT. (c) Rate of oxygen uptake in ex-vivo perfusion measured every 30 minutes and normalized to liver weight. (d) Total volume of bile produced during 6 hours of NMP, compared across the 90-min WI control, 90-min WI vehicle control and 90-min WI CEPT-treated groups. Livers treated with CEPT secreted small amounts of bile, while there was no bile production by the two other control 90-min WI groups. Fresh control livers, excluded from the graph, produced 3.03±0.76 mL of bile.

ALT and AST enzyme levels in the perfusate (Figs. 2a and 2b) serve as markers of hepatocellular injury. In CEPT-treated livers, AST levels (Fig. 2a) rose in the first 2 hours of perfusion, but then decreased and stabilized. In contrast, the 90-min WI control and vehicle control livers exhibit an accumulation of AST for the entire duration of perfusion. ALT levels in the perfusate (Fig. 2b) follow a similar trend, although the difference among experimental groups was not as large. The differences observed between the ischemic control groups and the CEPT treatment group were not found to be statistically significant (P > 0.05). Fresh control livers that did not undergo the additional 90-min warm ischemia maintained close-to-zero ALT and AST levels throughout the 6-hour period of NMP.

Oxygen uptake and bile secretion as markers of viability and function (Fig. 2c and 2d) are both severely affected by the extended duration of WI in the subjected groups. Oxygen uptake decreased in the WI Control, Vehicle Control and CEPT-treated groups, but remained relatively stable in fresh control livers. The two ischemic groups that were not treated with CEPT completely ceased to produce bile, while CEPT-treated livers still secreted a very small amount of bile (P < 0.0001).

### Liver functionality during reperfusion

**Figure 3:**
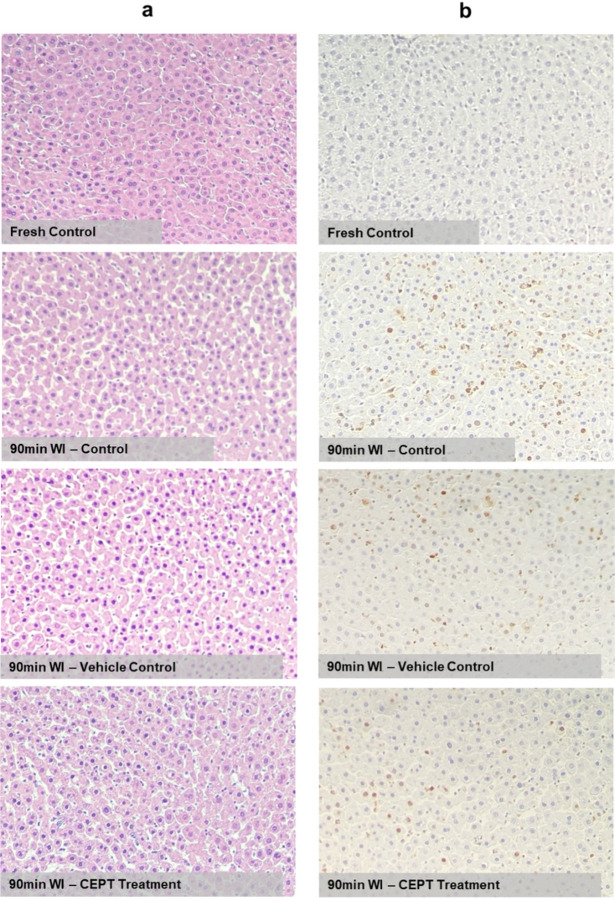
Morphology of ischemic hepatic tissue after 6 hours of CEPT treatment. (a) Light microscopy images of liver tissue collected at the end of NMP reveal lower frequency of cellular shrinkage in CEPT-treated samples. (b) Light microscopy images of TUNEL-stained liver tissue show a reduction in DNA fragmentation after treatment of ischemic livers with CEPT.

**Figure 4:**
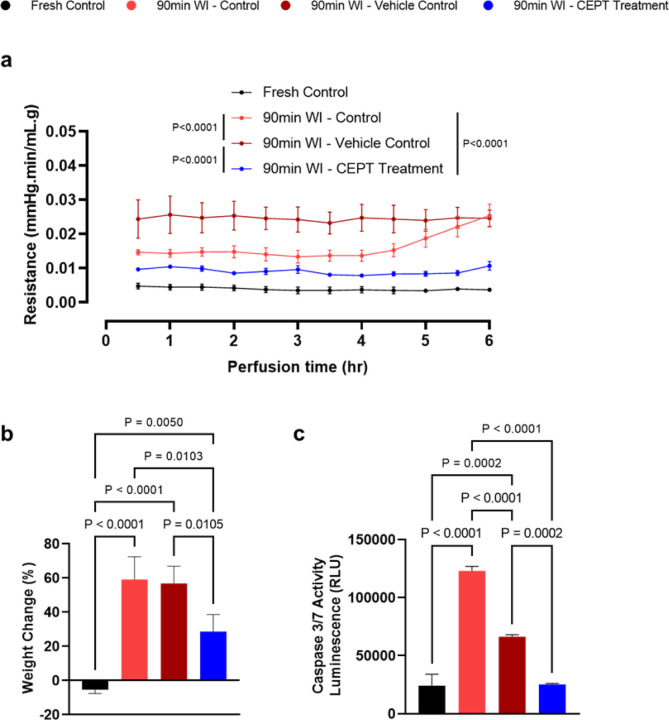
Treatment with CEPT may improve endothelial dysfunction following warm ischemia-reperfusion injury. (a) Vascular resistance of the portal vein. CEPT-treated livers exhibited the lowest vascular resistance among all warm ischemic groups. (b) Liver weight change after 6 hours of NMP. CEPT-treated livers show the lowest amount of weight gain on average among all warm ischemic groups, suggesting lower endothelial damage and edema. (c) Caspase 3/7 activity measured in flash-frozen liver samples collected at the end of NMP. Similar to the fresh control group, CEPT-treated livers exhibited significantly lower caspase activity than both the 90-min WI control and 90-min WI vehicle control livers.

Pathology slides were analyzed through H&E (Fig. 3a) and TUNEL staining (Fig. 3b). While the ischemic groups little differences in overall morphology, the TUNEL staining results suggest that livers treated with CEPT show a slight reduction of apoptosis when compared to the vehicle control group, and a more significant reduction when compared to the WI control group receiving plain media during reperfusion. As expected, the fresh control slides present limited to no apoptosis. The CEPT-treated warm ischemic livers showed significantly and consistently lower vascular resistance in the portal vein (Fig. 4a) compared to the two WI control groups (P < 0.0001), suggesting improved microvascular function following reperfusion; fresh control livers exhibited steadily low vascular resistance throughout the perfusion. As shown in Fig. 4b, both the 90-min WI control and vehicle control groups similarly experienced a significantly higher level of edema than the CEPT-treated group (P = 0.0103 and 0.0105 respectively), representing reduced microvascular damage and reaffirming the findings in Fig. 4a. As expected, fresh control livers showed a small amount of weight loss. Caspase 3/7 activity measured in hepatic tissue samples collected and flash frozen immediately following the 6-hour NMP is shown in Fig. 4c. CEPT-treated livers had significantly lower levels of expression compared to the 90-min warm ischemia (P < 0.0001) and vehicle (P < 0.0002) control groups, with no statistical difference with the fresh control livers.

## Discussion

Pan-caspase inhibition through Emricasan treatment was previously shown to mitigate inflammatory responses in discarded human livers in an NMP setting^8^, setting a promising precedent for targeting non-inflammatory apoptotic pathways as a means of improving marginal organ function. The goal of our study was to test a biochemically similar yet more comprehensive approach in the context of ischemia reperfusion injury. Using extensive high-throughput screening methods, a recent study developed a small molecule cocktail abbreviated as CEPT, with Emricasan as one of the components, which was optimized to improve viability of human pluripotent stem cells (hPSCs) and their progeny^7^; despite the clear difference between the functionality of hPSCs and liver cell types, the CEPT cocktail’s general mechanisms of action targeting promotion of cell growth, protein synthesis and stabilization of cellular structures led us to hypothesize that it could prove effective against reperfusion injury in warm-ischemic livers. We have shown that treatment of warm ischemic rat livers with the CEPT cocktail can result in amelioration of ischemia-reperfusion injury by demonstrating improvement of endothelial dysfunction, lower expression of hepatocellular injury markers and partial recovery of bile production.

Histological analysis of hepatic tissue after 6 hours of recovery NMP reveals slight morphological improvement after CEPT treatment, namely reduction of hepatocyte shrinkage, a characteristic phenotype associated with apoptotic hepatic tissue^9^. Along with the lower number of TUNEL-stained nuclei and significantly reduced levels of Caspase 3/7 expression in CEPT-treated tissue, this confirms effective hepatocellular uptake and biochemical impact of the cocktail under our perfusion conditions and duration. This is further validated by a comparison of ALT/AST concentration trends across the experimental groups that, although not statistically significant, points to an improved recovery in CEPT-treated livers; over the course of the 6-hour NMP both ALT and AST concentrations in the CEPT-treated group stabilize, contrasted by a relatively consistent rise in the warm ischemia and vehicle controls. The extended duration of ischemia in our study design leads to severe damage in the biliary epithelium, as is evident by the complete cessation of bile secretion in the warm ischemic control groups and supported by the literature^10^, however the slight recovery of bile production in the CEPT-treated livers could mean that the interference of apoptotic pathways prevent further cascading of injuries not only to hepatocytes, but also cholangiocytes, whose populations are known to drop by an acute increase in apoptosis following reperfusion^11^. This hypothesis is backed by our pre-existing knowledge that prolonged periods of warm ischemia cause aggravated biliary injury through initiation of apoptosis in bile duct epithelium^12^.

While it has been demonstrated that pan-caspase inhibition leads to amelioration of portal hypertension in chronically diseased livers, cirrhosis in specific^6^, its efficacy had not been previously tested in livers subjected to ischemic damage. Interestingly, vascular resistance in the portal vein was a major area of difference across our experimental groups. CEPT-treated livers expressed lower portal resistance throughout the NMP period than both other ischemic groups, while the vehicle control sustained the highest; following the same trend, weight gain was lowest when the ischemic liver was treated with CEPT, proposing a highly likely connection between the effects of the anti-apoptotic cocktail and preservation of endothelial integrity in reperfusion. This can be explained by a previously unexpected hypothesis, that despite signs of recovery in the liver parenchyma, CEPT may have a more direct, profound, and fast-acting effect on the hepatic sinusoids. Furthermore, considering that primary hepatocytes account for the majority of hepatic tissue by population (60–65%)^13^ and therefore its highest metabolic demand, this can provide a rationale for the absence of any significant recovery in overall oxygen consumption of warm ischemic livers when treated with CEPT.

Our data shows an overall improvement in viability and function of ischemic male rat livers following reperfusion as a direct result of exposure to the anti-apoptotic, survival-enhancing CEPT cocktail. While further biochemical and genetic analyses are required to map out the extent of CEPT’s effects on various hepatic cell types, and the metabolic, signaling and biosynthetic pathways being impacted by it, apoptosis appears to play a more substantive role in IRI than previously understood. Moreover, the goal of this study was not to elucidate the mechanism of action of each CEPT component in liver cells, but to investigate whether its previously shown effects are translatable in hepatic ischemia. Therefore, further work is required to investigate therapeutic targets, and explore alternative components for optimization in liver.

## Supplementary Material

Supplement 1
